# One-Year Self-Reported Appetite Is Similar in Adolescents with Obesity Who Do or Do Not Undergo Sleeve Gastrectomy

**DOI:** 10.3390/nu14153054

**Published:** 2022-07-26

**Authors:** Vibha Singhal, Supritha Nimmala, Nazanin Hazhir Karzar, Miriam Bredella, Madhusmita Misra

**Affiliations:** 1 Neuroendocrine Unit, Massachusetts General Hospital, Harvard Medical School, 55 Fruit st, Boston, MA 02114, USA; snimmala@mgh.harvard.edu (S.N.); nhazhirkarzar@mgh.harvard.edu (N.H.K.); mmisra@mgh.harvard.edu (M.M.); 2 Division of Pediatric Endocrinology, Massachusetts General Hospital, Harvard Medical School, 55 Fruit st, Boston, MA 02114, USA; 3 MGH Weight Center, Massachusetts General Hospital, Harvard Medical School, 55 Fruit st, Boston, MA 02114, USA; 4 Department of Radiology, Massachusetts General Hospital, Harvard Medical School, 55 Fruit st, Boston, MA 02114, USA; mbredella@mgh.harvard.edu

**Keywords:** appetite, sleeve gastrectomy, adolescents, hormones

## Abstract

Background: With the growing prevalence of severe obesity in adolescents, sleeve gastrectomy (SG), a type of metabolic bariatric surgery (MBS), is increasingly being performed at a younger age. Data regarding changes in homeostatic and hedonic appetite following SG are conflicting in adults, with some studies showing no change and others showing a decrease in appetite. Data evaluating the effect of SG on appetite during adolescence, when appetite is more plastic, are currently lacking. Objective: To evaluate appetite changes one year after SG in adolescents with obesity vs. in non-surgical controls (NS). Methods: Thirty-nine subjects 13–21 years old with severe obesity were followed for a year; 19 underwent SG, and 20 were followed without surgery. Subjects had fasting blood tests for appetite-regulating hormones and completed a visual analog scale for appetite assessment (VAS). Results: The SG group had a decrease in body mass index (BMI) at one-year (baseline: 48.2 ± 1.7 kg/m^2^; one-year follow-up: 42.6 ± 1.0 kg/m^2^ (*p* ≤ 0.0001)). No within- or between-group differences were noted in the one-year change in appetite in the SG and NS groups. After SG, fasting ghrelin decreased (*p* ≤ 0.0001); however, no changes were noted in peptide YY (PYY) levels. Changes in one homeostatic appetite measure following SG were inversely associated with changes in fasting PYY (r = −0.583, *p* = 0.011). Appetite changes were not associated with weight loss or final BMI. Conclusions: There were no changes in appetite measures one-year after SG from pre-surgery levels in adolescents with obesity, and appetite changes were not associated with changes in BMI. It is important to evaluate the impact of long-term appetite changes, if any, on weight loss after SG.

## 1. Introduction

Appetite changes following metabolic and bariatric surgery (MBS) have been implicated as one of the primary reasons for the acute weight loss that follows surgery [1]. Appetite is regulated by a complex physiological system comprising neural and hormonal mechanisms and specific neurotransmitters [2,3,4,5], and alterations in both homeostatic and hedonic appetite following MBS have the potential to impact energy intake [6,7]. Many factors, such as age and sex, play essential roles in the regulation of appetite [8]. Hormones known to impact appetite include (but are not limited to) ghrelin and orexin, which increase appetite, and insulin, peptide YY (PYY), and glucagon-like peptide 1, which decrease appetite. Data suggest that adolescents and young adults demonstrate flexibility in the establishment of appetitive traits with significant potential for modulation due to persistent neuroplasticity [9,10,11,12]. Given their ability to modulate appetite at this younger age (compared with adults), and with the aim of understanding the mechanisms contributing to weight loss post-surgery in youth, it is essential to evaluate appetite changes that occur in adolescents after weight loss interventions, as observations in adolescents may not necessarily replicate observations in adults.

MBS is currently the most effective means of weight loss, which occurs because of multiple mechanisms, including decreased caloric intake and alterations in hormonal, neural, and nutrient pathways [13]. The use of MBS at younger ages is increasing due to the increased prevalence of severe obesity in adolescence and because of the paucity of other effective weight loss strategies in this age group [14]. Sleeve gastrectomy (SG) is the most commonly employed MBS in adolescents [15]. Data regarding appetite changes after SG in adults are conflicting; some studies suggest no change in appetite, while others suggest that decreases in appetite after surgery persist even after one year [16,17]. There are no data evaluating the effect of SG on appetite during the adolescent and young adult years when appetite is more plastic. In this study, we aimed to evaluate changes in homeostatic and hedonic appetite in adolescents with moderate to severe obesity who underwent SG (one year after surgery) compared with adolescents with obesity who did not undergo surgery. We further explored the relationship between these appetite changes and changes in specific orexigenic (ghrelin) and anorexigenic (PYY) hormones following SG. We hypothesized that one year after SG, in adolescents and young adults, there would be decreases in homeostatic and hedonic appetite, which would be associated with changes in these hormones.

## 2. Participants and Methods

The study was performed at the Translational and Clinical Research Center (TCRC) of our institution (Massachusetts General Hospital) and was approved by the Institutional Review Board of our institution. It was compliant with the Health Insurance Portability and Accountability Act. All subjects 18 years and older and parents/guardians of subjects younger than 18 years provided written consent, and all subjects 13–17 years old provided assent.

### 2.1. Participants

We enrolled 39 adolescents/young adults between 13 and 21 years old with moderate to severe obesity (BMI ≥ 35 kg/m^2^ or ≥120% of the 95th percentile BMI for sex and age (class II obesity) with the presence of at least one comorbidity, or BMI ≥ 40 kg/m^2^ or ≥140% of the 95th percentile BMI (class III obesity)). Nineteen participants underwent SG, and twenty were non-surgical controls (NS) with similar BMIs. In the SG group, all participants had class III obesity, and only 1 participant had class II obesity. In the NS group, 14 participants had class III obesity, and 6 participants had class II obesity. Participants in the surgical arm of the study were recruited from centers performing MBS, and the choice of surgery was based on a combined decision-making process that included the treatment team and the patient. Non-surgical controls were followed without any intervention over the 12-month duration of the study. Because the primary endpoint of the main grant was related to bone endpoints, the exclusion criteria included a history of medical disorders and medications known to affect bone metabolism (other than calcium, vitamin D, and hormonal contraception), untreated thyroid dysfunction, smoking >10 cigarettes/day, substance abuse according to DSM-5, pregnancy, and lactation.

### 2.2. Study Protocol

Following a screening visit to confirm eligibility, study visits were performed at baseline (prior to SG) and 12 months after surgery. Control (non-surgical) subjects were also examined at baseline and after 12 months. Surgical participants followed standard post-operative nutrition guidelines provided by their clinical team. Non-surgical controls were followed by their pediatricians/internists at a frequency based on provider recommendations with routine management, which included counseling regarding diet and exercise, sleep habits, and screen time. Both the groups—surgical and non-surgical—were given instructions to follow a regular diet, have adequate sleep the night before the study visits, and not indulge in excessive exercise activity the day prior. Participants came to the TCRC in the morning after an overnight fast for study evaluations. Each subject underwent a history evaluation, physical examination, 24 h food recall, and fasting blood tests for ghrelin and PYY, and they completed a visual analog scale (VAS) in the fasting state during study visits [18,19]. Post-prandial serum concentrations of ghrelin and PYY were also assessed at 15, 30, 60, 90, and 120 min following the consumption of a mixed meal (360 mL of Boost). The total area under the curve (AUC) from 0 to 120 min for the appetite hormones was calculated using the trapezoidal rule. We used the Paffenberger questionnaire to evaluate exercise activity and average hours of sleep per week during the 6 months preceding any study visit [20]. The 24 h food recall was administered by a trained Registered Dietician; all foods and beverages consumed over the past 24 h were recorded and analyzed using a food composition database. The reviewed intake data were collected, and recall data were entered directly into the NDSR (Nutrition Data System for Research, a computer-based software application developed at the University of Minnesota Nutrition Coordinating Center). Weight was measured in a hospital gown on an electronic scale to the nearest 0.1 kg, and height was obtained in triplicate using a standard stadiometer. Body mass index (BMI) was calculated using the formula weight (kg)/height (meter)^2^. 

### 2.3. Laboratory Measures

Blood samples were collected in the appropriate red or lavender topped tubes and Pefabloc tubes at our Institution’s TCRC and processed accordingly by the TCRC lab into 2 mL serum and plasma aliquots after centrifuging. For ghrelin assays, the plasma from the Pefabloc tubes was centrifuged and then transferred into HCL tubes before being aliquoted into cryovials. The aliquots were stored in −80 °C freezers and tested in a single batch for assay of appetite hormones. Serum aliquots were used to assay PYY, and plasma tubes with HCL additive were used for the ghrelin assay. Serum ghrelin concentrations were measured by an enzyme-linked immunosorbent assay (ELISA) (EMD Millipore Corporation, MO, USA; intra-assay coefficient of variation (CV) of 1.32%, inter-assay CV of 6.62%, and sensitivity of 50 pg/mL). PYY serum concentrations were also measured by an ELISA (Millipore Corporation (Linco Research), Billierca, MA, USA; intra-assay CV of 17–18%, inter-assay CV of 12–18%, and sensitivity of 10 pg/mL). 

### 2.4. Appetite Measurements

A validated appetite visual analog scale (VAS) was used to assess appetite in the fasting state. The VAS is a 100-mm-long scale that participants mark in response to specific questions (Supplementary Materials). The questions on the VAS assessed hunger; satiety; fullness; estimated prospective food consumption; and desire to eat something fatty, salty, sweet, and savory. Subjects were requested to make a vertical mark along each line that best matched how they felt at the time. Each score was determined by measuring the distance from the left end of the line to the mark [18,19].

### 2.5. Statistical Analysis

Statistical analyses were performed using JMP Statistical Discovery Software (Version 15). Data are reported as means ± standard error of the mean (SEM). Variables were assessed for their distribution, and appropriate tests were used to analyze group differences. Student’s *t*-test was used when data were normally distributed or when data approximated a normal distribution following log transformation, and the Wilcoxon rank sum test was used when log transformation was not feasible (e.g., for variables with data points that were ≤0). Within-group 12-month changes were assessed using the paired t-test for parametric data (including after log transformation when necessary) and the Wilcoxon signed-rank test for non-parametric data. We also evaluated changes in appetite after controlling for weight changes, as weight is known to impact appetite [21,22]. A two-tailed *p*-value less than 0.05 was considered significant, and a two-tailed *p*-value less than 0.1 but greater than/equal to 0.05 was interpreted as a trend. Because these were discovery (exploratory) analyses, type 1 error control for multiple comparisons was not considered. Spearman correlation analysis was performed to determine associations between changes in appetite measures and changes in appetite hormones, total caloric intake, and BMI. These data should be interpreted keeping in mind that appetite measures were not the primary endpoint of this study, and hence the power may be limited. However, a post hoc power calculation based on effect size and variability indicated that 395 participants would be necessary for the study results to be significant. These numbers are prohibitive in an adolescent bariatric surgery population, and the clinical significance may be doubtful when such a large number of participants is necessary to demonstrate significance. 

## 3. Results

The study sample was predominantly female (15 of 19 in the SG group and 16 of 20 in the NS group). None of the participants were on appetite-altering medications.

### 3.1. Baseline Characteristics

The SG and NS groups did not differ in age or sex. Baseline weight and BMI were higher in the SG group, as previously reported [23]. At baseline, there were no differences in total calories consumed or homeostatic (VAS questions 1–4) and hedonic (VAS questions 5–9) appetite between the two groups. The groups also did not differ in baseline measures of fasting serum concentrations or the AUCs for the orexigenic hormone ghrelin and the anorexigenic hormone PYY. (Table 1 and Table 2)

### 3.2. One-Year Change in Weight, Appetite, Appetite Regulating Hormones, Caloric Intake, and Activity

As expected, the SG group sustained a significant decrease in weight, BMI, and fat mass over the year. For both homeostatic (VAS questions 1–4) and hedonic (VAS questions 5–9) appetite, we found no within- or between-group differences in changes in appetite measures over a year, except that the measure of hedonic appetite (how strong is your desire to eat your favorite food) increased over time in the NS group but not the SG group. Additionally, we found no between-group differences in the appetite measures at the one-year time point. Fasting serum concentrations of ghrelin decreased, whereas PYY remained unchanged following SG. Similarly, the SG group demonstrated a decrease in the ghrelin AUC but no change in the PYY AUC compared with the NS group. There were within- and between-group reductions in total calories consumed (on a 24-h dietary recall) in the SG group vs. the NS group. We did not find any within or between group changes in physical activity (moderate and vigorous) or average hours of sleep over one year. (Table 1 and Table 2)

### 3.3. Associations between Changes in Appetite Measures and Hormones and BMI Changes 

In the SG group, there was a negative association between changes in fasting PYY levels and changes in one of the homeostatic appetite measures (How much do you think you can eat?) (r = −0.59; *p* = 0.011). No associations were observed between changes in fasting ghrelin or AUC ghrelin and AUC PYY over one year in the SG group. Furthermore, no associations were observed between changes in appetite measures and changes in BMI or final BMI after one year in these participants. 

## 4. Discussion

Our study showed that adolescents and young adults who underwent SG exhibited no significant differences in fasting homeostatic and hedonic appetite one year after surgery compared with adolescents and young adults with severe obesity who did not undergo surgery. These results, other than one measure of hedonic appetite that increased in the control group but not the surgical group over the study duration, contradicted our proposed hypothesis. To our knowledge, this is the first study to evaluate hedonic and homeostatic appetite changes after SG in adolescents, an age group in which appetitive behaviors exhibit plasticity [9,10,11,12]. Data from adult studies are conflicting; one study showed a significant reduction in appetite as early as four weeks after SG, which was maintained over the 12-month study period [16]. However, another study in adults reported contradictory results; no change in fasting hunger was observed after three months, although the study reported improved satiety after a standardized test meal [17]. A recent study showed a reduction in the reward value of sweet foods after SG in adolescents 12 weeks after surgery, which was less pronounced at 52 weeks, suggesting that appetite changes observed acutely after SG may be attenuated with time [24]. Our findings confirm that the overall changes in homeostatic and hedonic appetite that occur one year after SG in youth are similar to those in youth who do not undergo SG, except that the surgical group did not demonstrate an increase in one measure of hedonic appetite (How strong is your desire to eat your favorite food?), whereas an increase in this measure was observed in the non-surgical group after 12 months. The lack of differences in appetite seen between the SG group and the non-surgical group may suggest that the factors regulating appetite—hormonal, neural, and others—adapt to the post-surgical state after one year, returning toward pre-surgical levels.

Reductions in weight, BMI, lean mass, and fat mass in the SG group were similar to those reported in previous studies of adolescents undergoing SG [25,26,27]. Of note, the SG group had greater reductions in total caloric intake compared with the non-surgical group over one year, despite similar appetite changes. 

In addition to mechanical restriction post gastrectomy [28], changes in levels of the orexigenic hormone and anorexigenic hormones may contribute to alterations in appetite regulation and caloric intake [29,30]. We found significantly greater decreases in fasting and post-prandial levels of ghrelin in the SG group vs. the NS group, as reported in other studies after SG [16,31,32]. However, we did not find any association between changes in ghrelin levels and appetite changes, unlike a study in adults that suggested that decreases in ghrelin may contribute to an early decrease in appetite after surgery [33]. PYY levels, both fasting and post-prandial, remained unchanged in our cohort, as reported in another study in adults one year after SG [32]. Changes in fasting PYY levels were negatively associated with changes in one homeostatic appetite measure of hunger in the SG group, consistent with its anorexigenic effect. Although not seen in this cohort, some studies have suggested that PYY levels increase after SG and that this may be one of the gut hormones that modulates homeostatic appetite in these patients [16,17,31]. No associations were noted between measures of hedonic appetite and appetite hormones in our study, similar to a study by Bernard et al., which reported that not all adults undergoing SG had changes in their perceptions of fat and sweet stimuli and that these changes were not associated with changes in gut peptides, suggesting a more complex regulation of hedonic appetite [34]. Our data are preliminary and need further validation in larger studies of adolescents undergoing SG. 

Of note, we found no significant associations between changes in fasting homeostatic or hedonic appetite and changes in BMI. Lopes et al. showed that after gastric bypass, the rate of weight regain was higher in those with lesser appetite changes [35]. The current literature is limited in exploring the association between weight loss and weight regain after SG in youth, and this association needs further evaluation. 

Our study is not without limitations. First, this is a small sample with a short duration of follow-up. In addition, we used only the VAS to assess homeostatic and hedonic appetite (although the VAS is a well-validated measure of appetite). Further, there are other hormonal and neuronal pathways and neurotransmitters that regulate appetite that were not assessed in this study and merit future evaluation. Another limitation to consider is that the caloric intake is based on recall, and underreporting of caloric intake has been reported in populations with obesity [36,37]. Finally, this is one of the first studies exploring appetite changes in adolescents and young adults after SG, and it should be considered exploratory. Future studies with more frequent and longer-duration follow-ups are necessary to address some of the questions raised by this study. Further, we were not able to assess certain confounders of appetite, such as stress, sleep, and menstrual cycle phase in females.

## 5. Conclusions

In adolescents who had SG, there were no within- or between-group changes in homeostatic appetite over the year following surgery, but one hedonic appetite measure that increased over the year in the non-surgical group did not change in the SG group. This suggests that after one year, most immediate post-surgical changes in appetite reported in the literature are lost. While changes in appetite were not associated with changes in BMI over one year, it is important to evaluate the impact of the absence of long-term appetite changes on long-term weight loss in patients undergoing SG.

## Figures and Tables

**Table 1:** Changes in weight, body composition, and hormones in participants one year after sleeve gastrectomy vs. non-surgical controls.

Variable	Treatment	Baseline (SG *n* = 19, Controls *n* = 20)	One Year (SG *n* = 19, Controls *n* = 20)	One-Year Change	*p*-Value for within-Group Change	*p*-Value for between -Group Change
Weight (kg)	**SG** **Controls**	138.9 ± 6.3 * 116.7 ± 4.4 *	100.7 ± 6.9 115.7 ± 4.9	−38.2 ± 3.4 −1.1 ± 1.9	**<0.0001** 0.87	**<0.0001**
BMI (kg/m^2^)	**SG** **Controls**	48.2 ± 1.7 * 42.6 ± 1.0 *	34.7 ± 2.1 41.7 ± 1.3	−13.4 ± 1.1 −0.9 ± 0.7	**<0.0001** 0.59	**<0.0001**
**DXA Measures of Body Composition, Total Caloric Intake, and Activity**						
Fat Mass (kg)	**SG** **Controls**	67.4 ± 3.2 * 56.3 ± 2.4 *	40.7 ± 3.2 55.2 ± 2.7	−26.7 ± 3.3 −1.1 ± 1.2	**<0.0001** 0.40	**<0.0001**
% Fat Mass	**SG** **Controls**	49.6 ± 1.1 47.6 ± 0.9	40.0 ± 1.8 46.5 ± 1.0	−9.6 ± 1.5 −1.1 ± 0.5	**<0.0001** 0.99	**<0.0001**
Lean Mass (kg)	**SG** **Controls**	66.6 ± 2.5 60.0 ± 2.3	57.5 ± 2.2 61.1 ± 2.4	−9.1 ± 1.4 1.0 ± 0.6	**<0.0001** 0.99	**<0.0001**
% Lean Mass	**SG** **Controls**	49.2 ± 1.0 51.0 ± 0.9	58.3 ± 1.6 51.7 ± 1.0	9.1 ± 1.5 0.7 ± 0.6	**<0.0001** 0.27	**<0.0001**
Total Caloric Intake (Kcal)	**SG** **Controls**	1586.4 ± 196.1 1568.6 ± 163.6	1194.3 ± 80.3 1591.0 ± 122.3	−392.1 ± 179.4 22.3 ± 178.1	**0.03** 0.91	**0.004** **^a^**
Activity (h/week)	**SG** **Controls**	27.0 ± 5.3 28.3 ± 5.7	33.6 ± 4.6 27.2 ± 3.8	6.6 ± 6.5 −1.1 ± 6.8	0.35 0.76	0.95 ^a^
Average Sleep (h/week)	**SG** **Controls**	50.9 ± 2.5 54.9 ± 1.6	48.3 ± 2.4 48.8 ± 2.1	−2.6 ± 1.6 −6.1 ± 2.2	0.20 **0.02**	0.23
**HORMONES**						
Ghrelin (pg/mL)	**SG** **Controls**	204.3 ± 35.0 167.8 ± 26.8	36.0 ± 8.3 247.0 ± 35.5	−168.4 ± 33.4 79.2 ± 31.5	**<0.0001** 0.13	**<0.0001**
AUC Ghrelin (10^3^ pg/mL)	**SG** **Controls**	18.6 ± 2.2 17.3 ± 2.3	5.6 ± 1.3 16.7 ± 2.1	−13.0 ± 1.6 −0.6 ± 1.4	**<0.0001** 0.68	**<0.0001**
PYY (pg/mL)	**SG** **Controls**	91.2 ± 7.5 84.4 ± 7.4	83.2 ± 8.4 78.4 ± 7.8	−8.1 ± 6.5 −6.1 ± 8.3	0.18 0.47	0.85
AUC PYY (10^3^ pg/mL)	**SG** **Controls**	13.3 ± 1.0 11.1 ± 0.7	17.6 ± 2.8 11.7 ± 1.1	4.3 ± 3.2 0.6 ± 1.0	0.21 0.55	0.49

Data are presented as means +/− standard error of the mean (SEM). Significant *p*-values (*p <* 0.05) are in bold. Asterisk (*) indicates a variable that was significantly different at baseline between the two groups. ^a^ *p*-value for log-transformed data.

**Table 2:** Changes in homeostatic and hedonic appetite in participants one year after sleeve gastrectomy vs. non-surgical controls.

**Variable**	**Treatment**	**Baseline** **(SG *n* = 19,** **Controls *n* = 20)**	**One Year** **(SG *n* = 19,** **Controls *n* = 20)**	**One-Year Change**	***p*** **-Value for within-Group 1-Year Change**	***p*** **-Value for between-Group 1-Year** **Change**
**HOMEOSTATIC APPETITE**						
How hungry do you feel?	**SG** **Controls**	61.9 ± 4.6 53.6 ± 5.5	55.3 ± 5.7 51.8 ± 4.8	−6.6 ± 5.7 −1.8 ± 8.1	0.27 0.83	0.63
How much do you think you can eat?	**SG** **Controls**	61.7 ± 3.6 60.7 ± 4.8	56.0 ± 3.6 62.5 ± 4.0	−5.7 ± 4.6 1.8 ± 4.8	0.23 0.71	0.27
How full do you feel?	**SG** **Controls**	18.6 ± 5.3 20.7 ± 5.5	23.8 ± 5.5 25.9 ± 4.7	5.2 ± 6.6 5.2 ± 7.6	0.63 0.26	0.66 ^a^
How satisfied do you feel?	**SG** **Controls**	23.8 ± 4.7 25.7 ± 4.7	36.3 ± 4.6 36.5 ± 4.6	12.5 ± 6.1 10.9 ± 6.8	0.10 0.05	0.86
**HEDONIC APPETITE**						
How strong is your desire to eat your favorite food?	**SG** **Controls**	40.4 ± 6.7 35.1 ± 5.6	42.7 ± 6.1 51.7 ± 5.6	2.3 ± 7.5 16.6 ± 7.3	0.57 **0.03**	0.13 ^a^^±^
Would you like to eat something sweet?	**SG** **Controls**	55.5 ± 6.7 62.1 ± 7.7	54.3 ± 7.6 57.2 ± 6.1	−1.3 ± 6.8 −5.0 ± 11.2	1.00 0.59	0.08 ^a^
Would you like to eat something salty?	**SG** **Controls**	44.9 ± 5.7 50.2 ± 5.6	35.0 ± 4.6 46.7 ± 5.8	−10.0 ± 7.5 −3.5 ± 7.4	0.20 0.65	0.54
Would you like to eat something savory?	**SG** **Controls**	38.5 ± 4.8 37.5 ± 5.3	35.5 ± 20.6 41.7 ± 20.1	−2.9 ± 7.5 4.2 ± 7.1	0.70 0.56	0.49
Would you like to eat something fatty?	**SG** **Controls**	65.1 ± 5.9 50.9 ± 6.9	55.8 ± 6.2 55.1 ± 5.8	−9.2 ± 8.4 4.2 ± 9.7	0.29 0.67	0.31

Data are presented as means +/− standard error of the mean (SEM). Significant *p*-values (*p <* 0.05) are in bold. ^±^ Trend (*p* = 0.09) after controlling for changes in weight; ^a^ *p*-value for log-transformed data.

## Data Availability

The datasets used and analyzed during the current study are available from the principal investigators of the study on reasonable request.

## Supplementary Materials

The following supporting information can be downloaded at: https://www.mdpi.com/article/10.3390/nu14153054/s1. Visual analog scale for appetite measures: Supplementary File S1 (pdf format).

## Abbreviations

SG	Sleeve Gastrectomy
MBS	Metabolic Bariatric Surgery
NS	Non-Surgical
VAS	Visual Analog Scale
BMI	Body Mass Index
PYY	Peptide YY
DSM-5	Diagnostic and Statistical Manual of Mental Disorders, Fifth Edition
ELISA	Enzyme-Linked Immunosorbent Assay
CV	Coefficient of Variation
SEM	Standard Error Mean
TCRC	Translational and Clinical Research Center
AUC	Area Under Curve
