# Supplementary material for: One-Year Self-Reported Appetite Is Similar in Adolescents with Obesity Who Do or Do Not Undergo Sleeve Gastrectomy
Source: Nutrients. 2022 Jul 26;14(15):3054. doi: 10.3390/nu14153054 (PMC9331365; doi:10.3390/nu14153054)
Supplement: Supplementary file 1 [file nutrients-14-03054-s001.zip › nutrients-1796357-supplementary.pdf]

Additional File 1: Visual Analogue Scale for Appetite Measure

Question	Left anchor	Right anchor
**1. How hungry do you feel?** * must provide value	I am not hungry at all	I have never been more hungry
**2. How strong is your desire to eat your favorite food?** * must provide value	Not strong at all	Extremely strong
**3. How satisfied do you feel?** * must provide value	I am completely empty	I cannot eat another bite
**4. How full do you feel?** * must provide value	Not at all full	Totally full
**5. How much do you think you can eat?** * must provide value	Nothing at all	A lot
**6. Would you like to eat something sweet?** * must provide value	Yes, very much	No, not at all
**7. Would you like to eat something salty?** * must provide value	Yes, very much	No, not at all
**8. Would you like to eat something savory?** * must provide value	Yes, very much	No, not at all
**9. Would you like to eat something fatty?** * must provide value	Yes, very much	No, not at all

Each item: Change the slider above to set a response — reset
